# An adaptive toolbox approach to the route to expertise in sport

**DOI:** 10.3389/fpsyg.2014.00709

**Published:** 2014-07-08

**Authors:** Rita F. de Oliveira, Babett H. Lobinger, Markus Raab

**Affiliations:** ^1^Department of Applied Sciences, London South Bank University, LondonUK; ^2^Institute of Psychology, German Sport University, CologneGermany

**Keywords:** heuristics, expertise, sport, talent development, talent identification, cues

## Abstract

Expertise is characterized by fast decision-making which is highly adaptive to new situations. Here we propose that athletes use a toolbox of heuristics which they develop on their route to expertise. The development of heuristics occurs within the context of the athletes’ natural abilities, past experiences, developed skills, and situational context, but does not pertain to any of these factors separately. This is a novel approach because it integrates separate factors into a comprehensive heuristic description. The novelty of this approach lies within the integration of separate factors determining expertise into a comprehensive heuristic description. It is our contention that talent identification methods and talent development models should therefore be geared toward the assessment and development of specific heuristics. Specifically, in addition to identifying and developing separate natural abilities and skills as per usual, heuristics should be identified and developed. The application of heuristics to talent and expertise models can bring the field one step away from dichotomized models of nature and nurture toward a comprehensive approach to the route to expertise.

## INTRODUCTION

Most current theories of expertise are based on the principle that knowledge underlies performance (Ericsson et al., 2006). Specifically, Ericsson et al. (2006) suggest that the specific knowledge of experts arises through about 10.000 h of deliberate practice. More research studies have also shown that previous knowledge guides attention for accurate performance (e.g., Bilalić et al., 2010). Drawing on the importance of knowledge for performance, the heuristic approach focuses on how that knowledge can be effectively searched and a how solution implemented.

Heuristics are rules of thumb that allow fast and frugal decision-making. The concept of heuristics was introduced by Simon (1982) to explain how humans decide when they have limited resources. He proposed that behavior could only be understood through analyzing both the person and the environment where the behavior took place. The subsequent work of Gigerenzer et al. (1999) identified and tested specific heuristics in a number of different environments. For instance, they found the recognition heuristic whereby people choose the option they recognize over the option they do not recognize (Gigerenzer and Goldstein, 1999). Recently, the simple heuristics research program has been used in the context of sport. Raab (2012) showed that athletes use simple heuristics both to make decisions and to implement them in the sports environment. What is still lacking, however, is an understanding of how simple heuristics develop in the route to expertise.

### HEURISTICS AS CHARACTERISTICS OF EXPERTISE AND TALENT DEVELOPMENT

The topic of expertise has been gaining increased prominence in science and the media because researchers, practitioners, and laypeople wish to replicate the route to success in the most efficient way. In sport, and especially in team sports, experts are those with repeated top-level performance who can most efficiently resolve the situation put before them. The route to that level of expertise will no doubt have involved uncountable attempts some successful but many unsuccessful. Here we will argue that the developmental process is inherently non-optimal and non-linear, but that this is indispensable to acquire the highest levels of expertise. To say that athletes show optimal adaptation to various situations related to their sport does not equate to saying that their actions or behaviors are optimal, but rather that these are cost-effective actions or behaviors. This is especially the case where performance involves fast decision-making. The best decision is not the optimal decision per se, but the one that can solve the current situation well enough and fast enough (Simon, 1982; Raab et al., 2009; Todd et al., 2012). It is important to qualify what is meant by optimal performance so that efforts to identify and develop talented athletes are geared toward functional (not optimal) decision-making. The route to expertise starts by demonstrating a talent. Talent identification is the process of recognizing the potential of an athlete to excel in a particular sport. Talent development, on the other hand, is the process by which an athlete can realize that potential, which includes benefiting from the most appropriate learning and training environments (Vaeyens et al., 2008).

### CLASSICAL MODELS IN TALENT DEVELOPMENT: NATURAL ABILITIES AND NURTURE

Natural abilities together with environmental and intrapersonal catalysts are, according to Gulbin et al. (2010), the non-random factors contributing to the developmental process which leads to specific competencies. The distinction between natural abilities and catalysts reflects current views on talent identification and development which show a facet of the nature vs. nurture debate (e.g., Baker et al., 2003; Epstein, 2013). The focus of this debate is on general natural abilities and environmental factors that lead to general competencies (cf. categorizations by Gagné, 1999). For instance perceptiveness and coordination are considered general natural abilities and thought to influence talent development. Athletes are often identified on general perceptual or motor skills, and their development often focuses on these general abilities. Paradoxically, researchers and practitioners agree that expert athletes specialize in their sport and that a general skill is not sufficient for expert performance. One of the marks of expertise is the use of unique solutions to solve situations in the playfield and in other situations related with the sport. In other words, expert athletes are characterized by their optimal adaptation to all things related with their sport, including effective decision-making *in situ* rather than by general abilities.

## THE HEURISTICS APPROACH TO TALENT DEVELOPMENT

A useful framework to understand unique adaptations to new situational contexts is the heuristic approach. Athletes use heuristics or rules of thumb which are specific to the type of situation and can be used rapidly without much cost. The development of heuristics occurs within the context of the athletes’ natural abilities, but also their past experiences, developed skills, and situational contexts. It does not pertain to any of these factors separately, instead, heuristics pertain to the repertoire of the athlete and it is our contention that talent identification methods and talent development models should be geared toward the assessment and development of heuristics. This can be done by improving the efficiency in the use of an existing heuristic (e g., calibrating the heuristic to more valid cues), or learning which heuristic fits best with a specific environment (e g., Gigerenzer and Gaissmaier, 2011). The heuristics repertoire consists of psychological, neurophysiological, and perceptual-motor adaptations (Raab et al., 2009; Raab, 2012; Todd et al., 2012). Each heuristic is used for specific situations in much the same way as a hammer is used for nailing pictures but not for cutting branches. By definition a heuristic is composed of at least three building blocks. They are *search rules*, *search-stopping rules*, and *decision rules.* Within sports a fourth building block has been proposed which deals with the *execution rules*. We will expand on these.

*Search rules* include two kinds of search: search for information cues and search for alternatives. In most ball games the alternatives are fixed (i.e., players can only either pass, dribble, throw, etc.) so the athlete searches for information cues to decide on which of these actions to use. While novice athletes may search for information cues randomly, expert athletes can directly use the information cues with the highest validity (de Oliveira et al., 2009; Esteves et al., 2011). When the alternative actions are not specified, then search rules for the action itself must be generated. In these cases the task characteristics specify whether it is most advantageous to broaden or limit the search. For instance in chess it is advantageous to broaden the search for options (Bilalic et al., 2009), whereas in more time-pressured sports it may be best to narrow the search for options (Raab and Johnson, 2007).

*Search-stopping* rules are the rules by which one stops searching for information cues or alternative actions. Classical models presumed that there was a way to compute the optimal stopping point where the costs of further search would exceed its benefits. However, to say that athletes show optimal use of heuristics does not equate to saying that their actions or behaviors are optimal, but rather that these are cost-effective actions or behaviors. This means that an expert athlete knows when the search for information cues must stop and will use whatever information was gathered to make the decision in due time. This also means that novice athletes must be placed in situations that potentiate their search for the most valid information cues, and must also be placed in situations where decisions must be made based on low-quality information cues.

*Decision rules* describe how a decision is made after search has been stopped. Decision rules define how the available information is used to make a decision. Psychology has a tradition of assuming that intelligent behavior implies weighting and combining information cues (e.g., multiple linear regression models), but the research on fast and frugal heuristics has shown that frequently less is more. For instance, the recognition heuristic is a decision rule whereby the option chosen is simply based on one valid cue that point to one option and not to an alternative option (Gigerenzer and Goldstein, 1999). Again, expert athletes have the heuristic repertoire to make the best decisions the fastest, whereas novice athletes must be placed in situations that build up their repertoire.

*Execution rules* address questions like what action to carry out and how to execute it as already described for decision processes (Raab et al., 2005). Those rules are based on individual experience (Raab and Johnson, 2007). Athletes should be exposed to situations that force them to decide between options to learn execution criteria and build heuristics for various situations.

These rules are the building blocks of heuristics and they can help explain how athletes develop their expertise in terms of decision-making and problem-solving which are key competences in expert performance.

### HEURISTICS IN THE ROUTE TO SPORT EXPERTISE: BUILDING AN ADAPTIVE TOOLBOX

Heuristics are domain-specific and can be used to formally describe the link between natural and nurtured characteristics. In fact heuristics are neutral in the nature vs. nurture debate because they can be learned but they can also be available at birth (cf., Baker et al., 2003, 2012). As an illustration, very small children will naturally cluster around a ball. The building blocks of the heuristic used might be: search for ball, stop searching when the ball is found, decide to move closer to the ball. This behavior continues until they learn that exploring the space increases the chance of receiving the ball. Here they may change the rules of the existing heuristic into: search for space in relation to the ball, stop when space is found, and decide to move the space. Again, this behavior will continue until they learn that not only space but also the defensive players are important in receiving the ball. Here they may again change the rules of the existing heuristic into: search for the space in relation to the ball and the defensive player, stop when you found it decide to move to the space.

Heuristics are also specific to the sportive situations that the athlete encounters. Therefore, nature and nurture, which are normally described separately in models of talent identification and development, can instead be described as heuristics. This would allow future research on expertise to conceptually integrate phylogenetic (natural abilities), ontogenetic (development) and situational factors. As an illustration, take the phenomenon of less-is-more for situations where there is an abundance of cues. We explore how athletes with different natural abilities (John and Mary) may become experts in their sport. John may have a natural ability to focus on a small number of cues and use those cues to maximal advantage. He will be identified as talented because of his consistent results in particular situations (rather than his creativity). During development, John may specialize in the use of those cues and become an expert in using them and hence build a narrow repertoire of expertise. Provided these are the most valid cues for the sports situation, John will also be an expert in his sport. If the sport offers a lot more variety, however, John will need to benefit from a varied training program that forces him to explore and use other cues for other situations. Mary, on the other hand, may have a natural ability to focus on a large number of cues and will therefore use various combinations of cues. She might be identified as talented because of her creative solutions (rather than her consistent results). During development, Mary may learn which cues are most valid to which situation and hence build a broad repertoire of expertise. Provided a number of cue combinations is required for the sports situation, Mary will also be an expert in her sport. If the sport offers little variety, however, Mary will need to benefit from a specialized training program that forces her to use the most valid cues.

## CONCLUSION

The heuristics approach to expertise is useful because it takes into account the natural abilities and development of the athlete, as well as the situations posed by the sport and the training environment. It partly addresses the eternal nature vs. nurture debate and can provide suggestions for training programs which aim at developing the individual natural abilities of athletes by providing adequate sports situations. This is currently being developed in the area of decision-making (Marasso et al., accepted). The practical application of heuristics to talent identification and talent development models will bring the field one step away from dichotomized models and toward a true comprehensive approach to the route to expertise. Future research can use the heuristics approach to investigate how the route to expertise sometimes deviates from the mainstream to create novel solutions. For instance, new techniques like the Fosbury flop in track-and-field and the Tsukahara’s vault in gymnastics (Bar-Eli et al., 2008) highlight alternatives found by expert athletes who did not fit a standard model of talent.

## Conflict of Interest Statement

The authors declare that the research was conducted in the absence of any commercial or financial relationships that could be construed as a potential conflict of interest.
